# Hyperuricemia Reflects Cardiometabolic Burden Rather than Left Ventricular Systolic Dysfunction Across Heart Failure Phenotypes: Insights from a Real-World Acute Heart Failure Cohort

**DOI:** 10.3390/jcm15145719

**Published:** 2026-07-21

**Authors:** Raluca Ibănescu, Sebastian Ciurescu, Roxana Buzaș, Anda Gabriela Militaru, Diana-Alexandra Mîțu, Loredana Nicoleta Ionică, Iacob-Daniel Goje, Ciprian Rachieru, Paul-Gabriel Ciubotaru, Vlad Sabin Ivan, Daian-Ionel Popa, Daniel-Florin Lighezan

**Affiliations:** 1 Advanced Cardiology and Haemostaseology Research Center, “Victor Babeș” University of Medicine and Pharmacy, No. 2 Eftimie Murgu Square, 300041 Timișoara, Romania; raluca.ibanescu@umft.ro (R.I.); militaru.anda@umft.ro (A.G.M.); diana-alexandra.mitu@umft.ro (D.-A.M.); ionica.loredana@umft.ro (L.N.I.); daniel.goje@umft.ro (I.-D.G.); ciprian.rachieru@umft.ro (C.R.); ivan.vlad@umft.ro (V.S.I.); dlighezan@umft.ro (D.-F.L.); 2 Department V, Discipline of Medical Semiology I, “Victor Babes” University of Medicine and Pharmacy, No. 2 Eftimie Murgu Square, 300041 Timisoara, Romania; 3 Emergency Municipal Clinical Hospital, 300254 Timișoara, Romania; daian-ionel.popa@umft.ro; 4 Doctoral School, “Victor Babes” University of Medicine and Pharmacy, 300041 Timisoara, Romania; 5 Research Center for Medical Communication, Victor Babes University of Medicine and Pharmacy, 300041 Timisoara, Romania

**Keywords:** acute heart failure, serum uric acid, left ventricular ejection fraction

## Abstract

**Background/Objectives:** Acute heart failure (AHF) is a leading cause of global morbidity, necessitating the search for reliable biomarkers to guide risk stratification. While hyperuricemia is highly prevalent in heart failure patients and historically considered a potential “cardiotoxin”, contemporary evidence suggests that it may function as a surrogate biomarker for cardiometabolic distress rather than an independent driver of myocardial dysfunction. This study aimed to determine whether serum uric acid acts as an independent predictor of left ventricular ejection fraction (LVEF) in a high-risk cardiometabolic cohort. **Methods:** This retrospective, cross-sectional study analyzed 306 patients hospitalized for acute heart failure at a tertiary hospital in Timisoara, Romania. Data were analyzed using JASP (v0.96). Statistical methods included Shapiro–Wilk testing, Spearman’s rank-order correlation, and independent samples t-tests. Multivariable ordinary least squares linear regression and maximum likelihood logistic regression were employed to identify predictors of LVEF and diagnostic classification, adjusting for comorbidities and diuretic administration. **Results:** Hyperuricemia was present in 64.6% of the cohort. Multivariable linear regression demonstrated that while age, sex, and diuretic use significantly predicted LVEF variance, serum uric acid did not (*p* = 0.356). Furthermore, logistic regression confirmed uric acid possessed no diagnostic utility in differentiating heart failure phenotypes (OR = 0.995; *p* = 0.973). **Conclusions:** Serum uric acid is not an independent predictor of reduced ejection fraction in acute heart failure patients. Elevated uric acid appears to be a biochemical shadow of generalized metabolic distress, systemic congestion, and the pharmacokinetic effects of diuretic therapy rather than a pathological driver of contractile dysfunction.

## 1. Introduction

Acute heart failure (AHF) remains a leading cause of global morbidity, hospitalization, and mortality, imposing an immense burden on healthcare systems worldwide. Given the pronounced clinical heterogeneity of AHF presentations, the rapid identification of underlying pathophysiological mechanisms is crucial for optimizing patient trajectories. Consequently, there is an ongoing, intensive search for accessible, reliable circulating biomarkers capable of unmasking subclinical hemodynamic distress, guiding risk stratification, and directing precision therapies [1]. While established markers have transformed clinical pathways, identifying low-cost, ubiquitous biochemical indices to further capture the complex multisystemic nature of AHF remains a clinical priority.

In this context, serum uric acid has emerged as a subject of sustained scientific scrutiny. Hyperuricemia is overwhelmingly prevalent in the heart failure demographic, frequently exceeding background population rates and correlating with adverse long-term outcomes [2]. This elevated prevalence is not isolated to heart failure; population-level data from the SEPHAR III study demonstrate that hyperuricemia is particularly frequent among elderly hypertensive patients, with serum uric acid levels significantly higher in individuals over 65 years compared to younger adults, independent of renal function [3]. However, the exact pathophysiological role of uric acid in the failing myocardium remains highly controversial [4]. A historical body of literature proposed the “cardiotoxin hypothesis”, positing that uric acid independently drives oxidative stress, endothelial dysfunction, and myocardial apoptosis, thereby directly impairing left ventricular systolic function. The molecular substrate of this hypothesis involves the activation of pro-inflammatory pathways, including nuclear transcription factor kappa B (NF-κB), which modulates cytokine expression and endothelial dysfunction in the context of acute cardiovascular disease [5]. Conversely, a contemporary paradigm shift, supported by the clinical failure of large-scale randomized controlled trials testing urate-lowering therapies, suggests that uric acid is merely an innocent bystander [6]. Under this model, hyperuricemia does not mechanically depress contractility, but rather serves as a surrogate biochemical shadow reflecting a global cardiometabolic burden, intense systemic venous congestion, and renal hypoperfusion.

A critical methodological gap in prior studies was the failure to decouple uric acid from the confounding effects of concurrent cardiometabolic comorbidities and the iatrogenic hyperuricemia induced by loop diuretic therapy via competitive proximal tubular inhibition [7].

Therefore, the objective of this cross-sectional study was to evaluate the association between serum uric acid levels and left ventricular ejection fraction (LVEF) at admission, and to determine whether hyperuricemia serves as an independent predictor of reduced systolic function in a cohort with a high cardiometabolic burden.

## 2. Materials and Methods

### 2.1. Study Design and Cohort Stratification

This research was structured as a retrospective, cross-sectional clinical investigation conducted at a tertiary hospital in Timisoara, Romania; data were gathered between January 2023 and December 2024. The primary objective was to evaluate the prognostic and diagnostic utility of admission serum uric acid in characterizing left ventricular ejection fraction (LVEF) among a phenotyped cohort of patients hospitalized with acute heart failure. The study protocol was developed and executed in accordance with standard ethical principles for medical research. Formal ethical sanctioning was explicitly granted by the Institutional Review Board under approval number [02/8 January 2026].

The study cohort was established by initially selecting patients hospitalized with a primary clinical diagnosis of acute decompensated heart failure who were admitted. Inclusion required the availability of a comprehensive baseline clinical, demographic, and biochemical profile obtained upon admission. From this initial pool, patients were excluded from the primary analysis if they lacked critical baseline laboratory evaluations, specifically admission serum uric acid measurements, or if they were missing the baseline echocardiographic data necessary to establish the primary dependent variable, namely the admission left ventricular ejection fraction. Furthermore, any patients who declined to provide informed consent or otherwise expressed a desire not to participate in the study were excluded, ultimately yielding the final analyzed cohort.

### 2.2. Clinical Data Acquisition and Patient Selection

The study evaluated a total cohort of 306 patients presenting with acute decompensated heart failure. A comprehensive clinical, demographic, and biochemical profile was retrospectively established for each participant. Primary data collection, organization, and curation were systematically performed utilizing Microsoft Excel.

The extracted variables encompassed core demographic parameters, including chronological age and biological sex. Detailed clinical and anthropometric data were recorded, capturing admission weight, NYHA functional classification (classes 1 through 4), and precise pharmacological regimens, specifically noting the administration of loop diuretics (furosemide) at both admission and discharge. A robust profile of concurrent cardiometabolic risk factors was documented, including smoking status, alcohol use, diabetes mellitus, dyslipidemia, and hypertension. Furthermore, key biochemical indices were extracted from baseline laboratory evaluations, specifically targeting serum uric acid (mg/dL), NT-proBNP (pg/mL), C-reactive protein (mg/L), and procalcitonin (ng/mL). The primary dependent variable recorded was the baseline left ventricular ejection fraction (LVEF), expressed as a percentage.

Echocardiographic assessment of left ventricular ejection fraction was performed using the biplane method of discs (modified Simpson’s rule), in accordance with the joint recommendations of the American Society of Echocardiography (ASE) and the European Association of Cardiovascular Imaging (EACVI). All echocardiographic studies were performed by certified cardiologists at the time of hospital admission. Based on the admission LVEF, patients were classified as heart failure with reduced ejection fraction (HFrEF; LVEF < 40%) or non-HFrEF (LVEF ≥ 40%), the latter encompassing both heart failure with mildly reduced ejection fraction (HFmrEF; LVEF 40–49%) and heart failure with preserved ejection fraction (HFpEF; LVEF ≥ 50%), in accordance with the 2021 ESC Heart Failure Guidelines. Hyperuricemia was defined as a serum uric acid level exceeding 5.5 mg/dL, applied as a uniform threshold across both sexes, reflecting the upper reference limit used by the participating institution’s clinical laboratory. NYHA functional class was assessed by the admitting cardiologist at presentation based on clinical symptoms, functional capacity, and signs of congestion at the time of acute decompensation. Cardiovascular risk was categorized as ‘High’ or ‘Very High’ in accordance with the 2021 ESC Guidelines on Cardiovascular Disease Prevention. The nosological composition of the cohort was predominantly characterized by hypertensive heart disease and valvular/mixed-etiology heart failure, consistent with the extreme hypertension prevalence (89.5%) and advanced age of the cohort. Due to the retrospective design, NT-proBNP and procalcitonin measurements were unavailable for a subset of patients (NT-proBNP: 238/306 [77.8%]; procalcitonin: 149/306 [48.7%]). Missing values were handled through complete-case analysis; no multiple imputation was performed. Sensitivity to missing data is acknowledged as a methodological limitation.

### 2.3. Statistical Analysis Framework

All statistical analyses and advanced mathematical modeling procedures were executed using JASP software, version 0.96. Initial data distributions were evaluated for mathematical normality utilizing the Shapiro–Wilk test. Continuous variables were characterized using standard measures of central tendency and dispersion, strictly presented as mean values accompanied by standard deviations, while categorical data distributions were expressed via absolute frequencies and valid percentages.

Inferential statistics were sequentially applied to evaluate the mathematical interactions between clinical covariates and myocardial function. For the assessment of non-normally distributed continuous variables and biomarkers, Spearman’s rank-order correlation metrics were employed. Group comparisons evaluating baseline clinical variables against normally distributed continuous parameters (such as LVEF) were assessed utilizing independent samples t-tests.

To isolate independent predictors of ejection fraction variance, advanced ordinary least squares multivariable linear regression models were constructed. These models sequentially evaluated granular clinical risk factors alongside aggregated cardiovascular risk parameters. The structural integrity of these linear models was strictly verified through multicollinearity diagnostics utilizing variance inflation factors (VIFs) to ensure an absence of mathematical artifact.

Categorical and binary variables (SEX, NYHA functional class, ALCOHOL use, FUROSEMIDE administration) were represented as binary predictors, which is a statistically appropriate and widely accepted approach for their inclusion in ordinary least squares multiple regression models.

Concluding the analytical trajectory, a maximum likelihood logistic regression model was computationally formulated to predict specific diagnostic classifications, namely the “Non-HFrEF” clinical phenotype. The overarching diagnostic performance and discriminatory capacity of this logistic regression model were robustly quantified utilizing comprehensive deviance measurements (McFadden and Nagelkerke R-squared metrics), overall correct classification rates, and operational sensitivity and specificity. Visual validation of predictive accuracy was conducted through receiver operating characteristic (ROC) curves to calculate the precise area under the curve (AUC).

## 3. Results

### 3.1. Baseline Characteristics

The study cohort comprised 306 patients, analyzed for both continuous and categorical baseline characteristics. The mean age of the cohort was 70.72 ± 10.87 years. The mean admission weight was 84.57 ± 16.29 kg, with a recorded weight change ranging between −5 and −2 kg. The primary independent biomarker, admission serum uric acid, demonstrated a mean of 6.490 ± 2.200 mg/dL among 289 valid cases. The dependent variable, admission left ventricular ejection fraction (hereafter referred to as EF_A), averaged 49.80 ± 12.88% (*n* = 305).

Additional biomarkers included NT-PROBNP (4863 ± 8171 pg/mL), C-reactive protein (9.709 ± 8.739 mg/L), and procalcitonin (1.793 ± 11.47 ng/mL). Shapiro–Wilk testing confirmed that, except for ejection fraction (*p* = 0.208); all continuous variables were non-normally distributed (*p* < 0.001).

**Figure 1:**
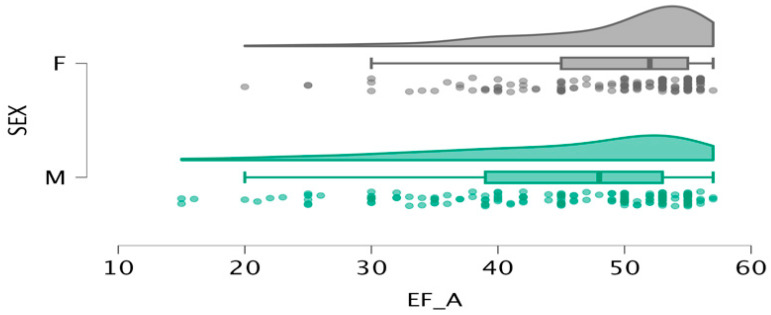
Raincloud plot illustrating the distribution of left ventricular ejection fraction (EF) at admission (EF_A), stratified by biological sex. Each panel combines a half-violin density plot (**top**), a boxplot displaying median and interquartile range (**middle**), and individual data points (**bottom**). Female patients (F, grey) demonstrate a more compact distribution with higher median ejection fraction values, while male patients (M, teal) exhibit a broader, right-skewed distribution extending toward higher ejection fraction values. These patterns are consistent with the known epidemiological predilection of older women toward heart failure with preserved ejection fraction (HFpEF). One observation was excluded due to missing data.

**Figure 2:**
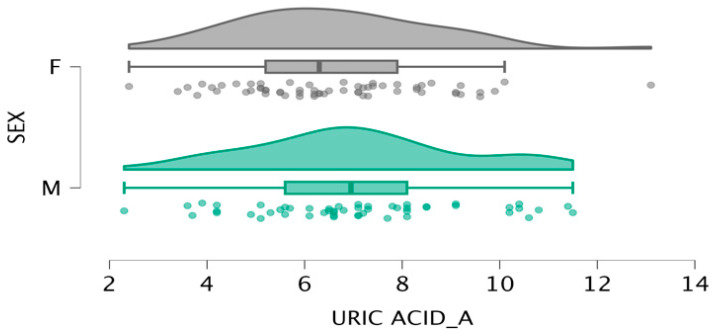
Raincloud plot illustrating the distribution of serum uric acid at admission (Uric Acid_A), stratified by hyperuricemia status. Each panel combines a half-violin density plot (**top**), a boxplot displaying median and interquartile range (**middle**), and individual data points (**bottom**). Patients classified as hyperuricemic demonstrate markedly elevated and more dispersed uric acid values compared to normouricemic patients, reflecting the high prevalence of hyperuricemia (64.6%) observed in this acute heart failure cohort. Fifteen observations were excluded due to missing data.

Categorical analysis revealed a relatively balanced sex distribution (51.6% male, 48.4% female), as displayed in Figure 1 and Figure 2.

Most patients presented with intermediate heart failure symptoms, specifically NYHA Class 2 (61.8%) and Class 3 (32.4%). Furosemide was administered to 78.3% of patients at admission and 77.9% at discharge. The cohort exhibited high prevalence rates for cardiovascular risk factors, notably hypertension (89.5%), dyslipidemia (84.6%), and diabetes mellitus (37.6%), with 71.6% of the total cohort classified as having a “Very High” cardiovascular risk. These findings are summarized in Table 1.

Echocardiographic phenotyping revealed that 61 patients (20.0%) met the criteria for HFrEF (LVEF < 40%), 75 patients (24.6%) had HFmrEF (LVEF 40–49%), and 169 patients (55.4%) had HFpEF (LVEF ≥ 50%). This distribution reflects the contemporary epidemiological landscape of acute heart failure admissions, wherein preserved ejection fraction phenotypes constitute the majority of hospitalizations, particularly in older, predominantly hypertensive cohorts.

### 3.2. Advanced Statistical Analysis

**Figure 3:**
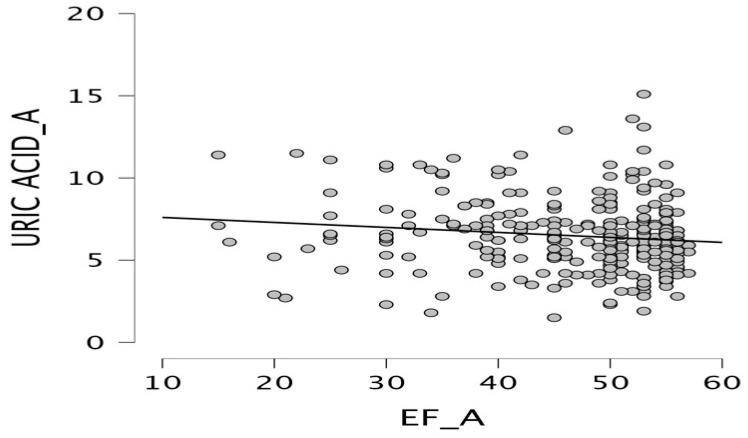
Scatter plot illustrating the relationship between serum uric acid at admission (Uric Acid_A) and left ventricular ejection fraction at admission (EF_A). Spearman’s rank-order correlation revealed a non-significant inverse association (rho = −0.107, *p* = 0.069), indicating the absence of a meaningful linear relationship between serum uric acid and systolic function in this cohort.

Non-parametric tests were required for biomarkers due to non-normal distributions. The correlations between EF and PROCALCITONIN (rho = 0.051, *p* = 0.536) and EF and URIC ACID (rho = −0.107, *p* = 0.069) were not statistically significant, as illustrated in Figure 3.

To further contextualize the inflammatory dimension of hyperuricemia, Spearman’s rank-order correlations were computed between serum uric acid and the two available inflammatory biomarkers. Serum uric acid demonstrated a weak positive association with C-reactive protein (rho = +0.117, *n* = 274) and a weak inverse association with procalcitonin (rho = −0.200, *n* = 86), neither of which reached a magnitude consistent with clinically meaningful co-linearity. These findings indicate that within this cohort, serum uric acid elevation is not primarily driven by systemic inflammatory activity, supporting its interpretation as a multi-factorial epiphenomenon of metabolic distress, congestion, and pharmacological exposure rather than a pure inflammatory surrogate.

**Figure 4:**
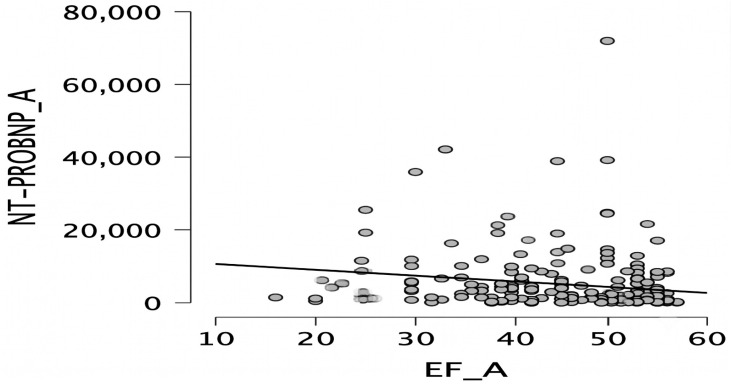
Scatter plot illustrating the relationship between NT-proBNP at admission (NT-PROBNP_A) and left ventricular ejection fraction at admission (EF_A). Spearman’s rank-order correlation demonstrated a statistically significant inverse association (rho = −0.282, *p* < 0.001), confirming that natriuretic peptide burden—rather than serum uric acid—serves as the clinically meaningful correlate of impaired systolic function in this acute heart failure cohort.

Spearman’s rank-order correlation revealed a statistically significant inverse correlation between ejection fraction and NT-PROBNP (rho = −0.282, *p* < 0.001), as illustrated in Figure 4.

A significant positive correlation was also observed between NT-PROBNP and PROCALCITONIN (rho = 0.284, *p* = 0.001). Furthermore, an independent samples t-test evaluating EF differences across baseline groups yielded a non-significant result (t = 0.828, df = 288, *p* = 0.409).

To isolate independent predictors of EF, two ordinary least squares multivariable linear regression models were constructed. As displayed in Table 2, the primary model (M1) incorporated granular clinical risk factors. The model was statistically significant (F = 6.553, *p* < 0.001), explaining 24.9% of the variance in EF (R^2^ = 0.249, Adjusted R^2^ = 0.211). Significant independent predictors of higher EF included AGE (*p* < 0.001), female SEX (*p* < 0.001), and ALCOHOL use (*p* = 0.024). Conversely, NYHA Class 3 (*p* = 0.041), NYHA Class 4 (*p* = 0.011), and FUROSEMIDE (*p* = 0.023) significantly predicted lower EF. URIC ACID was not a significant predictor (*p* = 0.356).

A secondary linear model replaced individual comorbidities with an aggregated cardiovascular risk parameter. This model was also significant (F = 6.799, *p* < 0.001, R^2^ = 0.223). Uric acid remained non-significant (*p* = 0.739), while AGE, SEX, NYHA 3, and NYHA 4 retained significance. Additionally, “High” cardiovascular risk emerged as a significant positive predictor of EF (B = 6.766, *p* = 0.028) relative to the baseline.

A maximum likelihood logistic regression model was utilized to predict the likelihood of a “non-HFrEF” diagnostic status (coded as 1). The full model significantly improved upon the null model (ΔX^2^ = 27.144, df = 99, *p* = 0.012), yielding a McFadden R^2^ of 0.227 and a Nagelkerke R^2^ of 0.327. Analysis of individual predictors demonstrated that AGE significantly increased the odds of non-HFrEF (OR = 1.099, 95% CI: 1.037–1.163, *p* = 0.001). NT-PROBNP was also statistically significant (*p* = 0.018), though its odds ratio approximated strictly 1.000 due to scale. Uric acid was not a significant predictor of the classification (OR = 0.995, *p* = 0.973). Multicollinearity was absent across all parameters (VIF ranges: 1.025–1.642) (Table 3).

**Figure 5:**
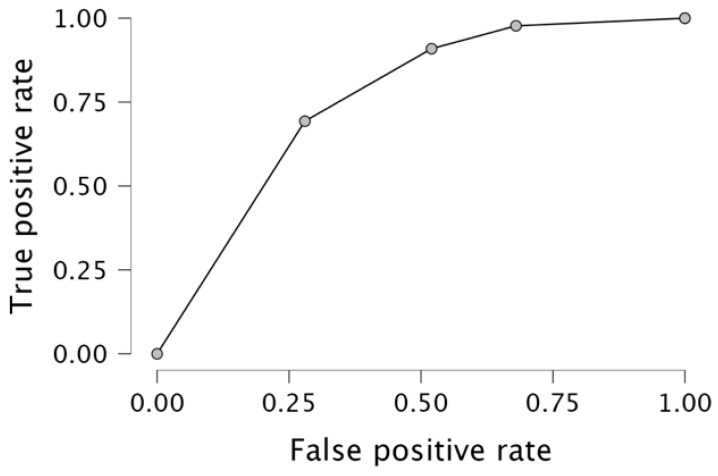
The ROC curve illustrates the diagnostic performance of the multivariable model in predicting the non-HFrEF status, achieving an area under the curve (AUC) of 0.804.

The logistic model demonstrated strong discriminatory performance (summarized in Table 4). Utilizing a 0.5 classification threshold, the model correctly predicted 85 out of 88 non-HFrEF cases and 9 out of 25 HFrEF cases. This resulted in an overall accuracy of 83.2%, a high sensitivity of 96.6%, and a specificity of 36.0%. The area under the curve (AUC) achieved a robust 0.804 (Figure 5).

## 4. Discussion

The primary objective of this retrospective, cross-sectional study was to evaluate the diagnostic and prognostic utility of admission serum uric acid (UA) in characterizing LVEF in a phenotyped acute heart failure cohort. The principal finding is that although hyperuricemia is highly prevalent in this setting, it does not independently predict reduced ejection fraction after adjustment for cardiometabolic and clinical covariates, consistent with the EXACT-HF investigators [8].

### 4.1. Principal Findings and Comparison with Existing Literature

Baseline characterization revealed an older cohort with substantial systemic metabolic derangement, consistent with Ndrepepa and Ezzat et al. [9,10]. Hyperuricemia affected roughly two-thirds of the valid sample, well above the general population rates. Extreme rates of concurrent cardiometabolic risk factors placed most patients in the “Very High” cardiovascular risk category, aligning with Borghi et al. [11]. The cohort also showed significant congestion, intermediate-to-severe symptoms, and heavy admission loop diuretic use, paralleling prior reports [12,13,14].

Despite this high prevalence, univariate correlations and group comparisons were non-significant. Multivariable linear regression confirmed the null finding: uric acid showed no significant association with LVEF and no multicollinearity, fulfilling the criteria of Kaufman and Guglin [15]. Instead, structural, demographic, and therapeutic variables drove LVEF variance—older age and female sex independently predicted higher ejection fraction, consistent with the predilection of older women for HFpEF [16,17], while severe symptoms and admission furosemide predicted lower ejection fraction, mirroring Virdis et al. [18].

Secondary modeling with a composite cardiovascular risk parameter corroborated these results: uric acid remained non-significant, while “High” cardiovascular risk was a significant predictor [19]. A logistic model predicting “Non-HFrEF” classification identified only age and NT-proBNP as significant determinants, consistent with the role of natriuretic peptides in HF phenotyping [20], and uric acid showed no discriminatory utility [21]. This aligns with the evolution of heart failure classification toward a multidimensional, LVEF-based framework [22] and with the limited independent physiological impact reported by Noman et al. [23].

The logistic subsample showed marked class imbalance (25 HFrEF vs. 88 non-HFrEF), yielding low specificity (36.0%) despite high sensitivity (96.6%); the high AUC (0.804) was driven mainly by age and NT-proBNP rather than uric acid. No threshold adjustment or class-weighting was applied, a recognized limitation.

Although earlier observational data linked elevated uric acid to adverse chronic heart failure outcomes—prompting its inclusion in staging systems like the MFH score—subsequent interventional evidence has undermined the cardiotoxin hypothesis. The SEPHAR III study confirms hyperuricemia tracks with cumulative cardiovascular risk burden rather than isolated myocardial dysfunction [3,5].

Rather than an active pathological driver, uric acid increasingly appears to be a biochemical marker of cardiometabolic distress, endothelial dysfunction, and renal hypoperfusion [24,25], consistent with evidence implicating inflammation and oxidative stress in cardiovascular remodeling [26,27] and with the NF-κB-mediated inflammatory framework of Buzas et al. [5], wherein uric acid is downstream rather than causal. Our findings align with this paradigm: the absence of an independent uric acid–LVEF association mirrors the failure of major trials testing xanthine oxidase inhibition.

The OPT-CHF trial [28] randomized 405 HF patients to oxypurinol or the placebo; despite lowering uric acid, LVEF and outcomes were unaffected. The EXACT-HF trial [8], in 253 hyperuricemic HFrEF patients given high-dose allopurinol, achieved a 3.5 mg/dL uric acid reduction without improving clinical status, exercise capacity, or LVEF. More recently, the ALL-HEART trial [6], in over 5700 patients with ischemic heart disease, found that up-titrated allopurinol did not improve cardiovascular outcomes, including HF hospitalization or death.

This dissociation between uric acid lowering and systolic improvement supports our regression findings. The cohort’s high prevalence of hypertension (89.5%), dyslipidemia (84.6%), and diabetes (37.6%) explains why uric acid did not independently predict LVEF: consistent with SEPHAR III, hyperuricemia clustered with hypertension and metabolic abnormalities rather than acting as an isolated pathological entity [3,29]. In such high-burden populations, myocardial variance is dominated by ischemic, hypertensive, and fibrotic remodeling, and the ESC-EORP registry analysis by Ambrosio et al. [7] similarly showed that hyperuricemia’s prognostic value diminishes after adjustment for age, chronic kidney disease, and metabolic syndrome.

### 4.2. Mechanistic Insights and the Cardiometabolic Phenotype

Understanding why uric acid fails to independently predict reduced ejection fraction requires considering its generation, transport, and excretion in acute decompensated heart failure. The hyperuricemia in 64.6% of our cohort reflects three overlapping drivers: (1) upregulated xanthine oxidoreductase activity under oxidative and hypoxic stress; (2) competitive inhibition of proximal tubular OATs by loop diuretics; and (3) splanchnic and renal venous congestion impairing urate excretion.

Uric acid is the terminal product of purine catabolism, catalyzed by xanthine oxidoreductase (XOR). Under tissue hypoxia and metabolic stress, XOR shifts from its dehydrogenase to its oxidase form (XO), as reviewed by Doehner et al. [30] in their EMPEROR-Reduced analysis; the conversion of hypoxanthine to xanthine and xanthine to uric acid simultaneously generates reactive oxygen species (ROS), including superoxide and hydrogen peroxide.

Contemporary mechanistic analyses, notably articulated by Packer [31], clarify that uric acid is a byproduct of XO-mediated ROS generation—not the active driver. The true mediator of myocardial depression is localized ROS production and myocardial ATP depletion, not the circulating urate anion. The failure of uric acid to predict LVEF in our regression model supports this concept: it is an innocent bystander, a biomarker of oxidative stress, not a direct participant in contractile dysfunction.

A further confounder is the high prevalence of furosemide use (78.3% at admission, 77.9% at discharge). Loop diuretic therapy is a well-established driver of iatrogenic hyperuricemia through a mechanism that undermines uric acid’s utility as an independent myocardial predictor, as explored by Doehner et al. [32] in the EMPEROR-Preserved context.

Because furosemide competes at these same transporters, high-dose loop diuretic therapy blocks tubular urate secretion, driving serum uric acid accumulation that is largely independent of myocardial state or LVEF.

In our multivariable regression analysis, furosemide administration emerged as a significant independent predictor of lower LVEF, whereas uric acid did not. This indicates that the requirement for loop diuretics, rather than uric acid itself, is the clinical correlate of reduced systolic function. The hyperuricemia measured in 64.6% of these patients therefore reflects, to a large extent, a pharmacokinetic consequence of decongestive therapy rather than an intrinsic myocardial process.

### 4.3. Clinical Implications

This study’s clinical implications are highly relevant to contemporary practice. Most importantly, the data argue against using admission serum uric acid as a surrogate for left ventricular systolic function. While uric acid remains a low-cost biochemical tool in acute heart failure, its utility lies in gauging systemic metabolic distress, cumulative diuretic burden, and cardiorenal congestion consistent with Min et al. [33].

Elevated uric acid (present in roughly two-thirds of the cohort) should not prompt suspicion of severely reduced LVEF. The logistic model predicting the non-HFrEF phenotype showed an odds ratio for uric acid of approximately 1, indicating hyperuricemia is distributed evenly across ejection fraction phenotypes. HFpEF patients are as likely to present with hyperuricemia—driven by age, diabetes, obesity, and inflammation—as HFrEF patients.

These findings also inform the debate on urate-lowering therapy (ULT). Because uric acid is not an independent driver of reduced ejection fraction, isolated urate reduction is unlikely to reverse systolic dysfunction—consistent with the neutral effect of high-dose allopurinol on ejection fraction, hospitalizations, and mortality in the trials above.

Notably, the most effective contemporary heart failure therapies modulate uric acid incidentally, with myocardial benefits occurring independently of urate-lowering effects. SGLT2 inhibitors (dapagliflozin, empagliflozin) reduce cardiovascular mortality across the LVEF spectrum while lowering uric acid; post hoc analyses of DAPA-HF [34], EMPEROR-Reduced [30], and EMPEROR-Preserved [30] showed uniform benefit across all uric acid tertiles, indicating urate reduction is a byproduct rather than the mechanism of cardioprotection.

Similarly, sacubitril/valsartan modestly lowers uric acid [35,36], yet its mortality and morbidity benefits are independent of baseline uric acid levels.

Treatment intensity and adherence to guideline-directed medical therapy remain closely tied to outcomes even in advanced disease [37]. Accordingly, hyperuricemia in a decompensated heart failure patient should prompt intensive GDMT optimization and reassessment of the decongestive regimen rather than allopurinol initiation, consistent with the epidemiological data of Thanassoulis et al. [21].

### 4.4. Study Limitations

Several limitations should be considered. First, the retrospective, cross-sectional design relies on a single biochemical and echocardiographic measurement at admission. As heart failure is dynamic and uric acid levels fluctuate with fluid status, diet, renal perfusion, and oxidative stress, a single time-point cannot capture its longitudinal trajectory or establish causality. The null finding does not exclude a prognostic role for uric acid; serial measurements would be needed to assess whether its dynamic changes predict systolic decline or adverse outcomes.

Second, loop diuretic use was modeled as a binary variable, without cumulative dose or furosemide-equivalent data, precluding full separation of iatrogenic urate retention from intrinsic hyperuricemia.

Third, creatinine and eGFR were not prospectively collected. Since renal dysfunction is the principal determinant of uric acid retention in heart failure, their omission may have allowed residual confounding; future studies should include eGFR as a mandatory covariate. The uniform 5.5 mg/dL hyperuricemia threshold, reflecting local laboratory reference values, may overclassify hyperuricemia relative to current sex-specific cut-offs. Sensitivity analyses with sex-specific thresholds are warranted.

Fourth, combining HFmrEF and HFpEF into one non-HFrEF category may obscure phenotypic differences. Stratified analyses would clarify whether uric acid—recently linked to right ventricular dysfunction [38]—associates differentially with specific HF phenotypes.

Fifth, only admission LVEF was assessed; diastolic parameters (E/e’, left atrial volume index) and right ventricular indices (TAPSE, RV fractional area change) were not evaluated, despite their contribution to acute heart failure and potential interaction with hyperuricemia. Uric acid has been proposed as a marker of right ventricular dysfunction in mechanical circulatory support patients [38]. Comprehensive echocardiographic phenotyping is needed in future work.

Sixth, missing laboratory values (NT-proBNP and procalcitonin) were handled by complete-case analysis; the absence of multiple imputation means that patients with incomplete biomarker data may differ systematically from those with complete data.

Finally, the cohort’s high cardiometabolic burden (89.5% hypertension, 84.6% dyslipidemia, 71.6% “Very High” cardiovascular risk), while representative of inpatient cardiology populations, creates a ceiling effect that compresses the independent variance attributable to a single biomarker. Lack of longitudinal follow-up further precludes assessing whether uric acid predicts gradual systolic decline over years rather than acute correlates.

## 5. Conclusions

The present study contributes to the growing body of evidence examining the cardiometabolic determinants of systolic dysfunction in acute heart failure, demonstrating that serum uric acid, despite its high prevalence in this population, does not emerge as an independent predictor of left ventricular ejection fraction following adjustment for relevant clinical and cardiometabolic covariates. These findings are consistent with the hypothesis that hyperuricemia in the setting of acute heart failure functions primarily as an epiphenomenon, reflecting the cumulative pathophysiological burden of cardiometabolic impairment, systemic congestion, renal dysfunction, and diuretic exposure, rather than exerting a direct mechanistic influence on systolic performance. Notwithstanding this observation, the robust and consistent association of hyperuricemia with multiple adverse clinical and metabolic characteristics underscores its potential utility as an integrated biomarker of disease complexity and cardiovascular vulnerability. Accordingly, the clinical significance of serum uric acid in heart failure may be better captured within a multimarker framework, in which it provides incremental contextual information rather than serving as a standalone indicator of myocardial dysfunction.

Future investigations employing prospective designs and comprehensive biomarker panels are warranted to further elucidate the independent prognostic contribution of serum uric acid and to advance phenotypic characterization and risk stratification in this high-risk population.

## Figures and Tables

**Table 1:** Clinical and demographic characteristics of the study cohort. Continuous data presented as mean ± standard deviation. Categorical data presented as frequency (valid percentage).

Variable	Metric/Category	Value
AGE (Years)	Valid N/Mean ± SD	306/70.72 ± 10.87
WEIGHT (kg)		306/84.57 ± 16.29
URIC ACID_(mg/dL)		289/6.49 ± 2.200
EF (%)		305/49.8 ± 12.88
NT-PROBNP (pg/mL)		238/4863 ± 8171
PCR (mg/L)		287/9.709 ± 8.739
PROCALCITONIN (ng/mL)		149/1.79 ± 11.47
SEX	Male/Female	158 (51.6%)/148 (48.4%)
HYPERURICEMIA STATUS	Normal/Hyperuricemia	103 (35.4%)/188 (64.6%)
NYHA	1/2/3/4	7 (2.3%)/189 (61.8%)/99 (32.4%)/11 (3.6%)
FUROSEMIDE	No/Yes	66 (21.7%)/238 (78.3%)
SMOKING		212 (69.3%)/94 (30.7%)
DIABETES MELLITUS		191 (62.4%)/115 (37.6%)
DYSLIPIDEMIA		47 (15.4%)/259 (84.6%)
HYPERTENSION		32 (10.5%)/274 (89.5%)
CARDIOVASCULAR RISK	High/Very High	46 (15.0%)/219 (71.6%)

**Table 2:** Significant coefficients and variables of interest in the primary linear regression model (M_1_) predicting EF. Non-significant predictors (PCR, SMOKING, DIABETES MELLITUS, DYSLIPIDEMIA, HYPERTENSION) are omitted from the display for brevity but were included in the model. VIF = variance inflation factor (values ~1.0 indicate no multicollinearity). Significance defined at alpha < 0.05.

Model Predictor	Unstandardized B	SE	Standardized β	t	*p*-Value	VIF
(Intercept)	29.04	7.081		4.101	<0.001	
URIC ACID	0.313	0.338	0.054	0.925	0.356	1.078
AGE	0.265	0.073	0.223	3.610	<0.001	1.141
SEX (F)	6.279	1.557		4.032	<0.001	1.113
NYHA (3)	−11.03	5.360		−2.057	0.041	
NYHA (4)	−16.41	6.409		−2.560	0.011	
ALCOHOL (Yes)	3.863	1.695		2.279	0.024	1.113
FUROSEMIDE (Yes)	−4.272	1.867		−2.288	0.023	1.134

**Table 3:** Key logistic regression coefficients for predicting non-HFrEF. Non-HFrEF (non-heart failure with reduced ejection fraction) is coded as 1. CI = confidence interval; SE = standard error. Significance (*p*-value) calculated via Wald test.

Predictor	Estimate	SE	Odds Ratio	95% CI (OR)	*p*-Value
(Intercept)	−3.900	2.586	0.020	0.000–3.216	0.131
URIC ACID	−0.005	0.146	0.995	0.747–1.325	0.973
AGE	0.094	0.029	1.099	1.037–1.163	0.001
NT-PROBNP	−0.000	0.000	1.000	1.000–1.000	0.018
NYHA (4)	−2.213	1.394	0.109	0.007–1.682	0.112

**Table 4:** Confusion matrix for logistic regression model. The classification cut-off value is strictly set to 0.5. HFrEF = heart failure with reduced ejection fraction.

Observed/Predicted	HFrEF	Non-HFrEF	% Correct
HFrEF	9	16	36.00%
Non-HFrEF	3	85	96.59%
Overall % Correct			83.19%

## Data Availability

The raw data supporting the conclusions of this article will be made available by the authors on request.

## Abbreviations

The following abbreviations are used in this manuscript:

**Abbreviation**	**Definition**
AHF	Acute heart failure
ARNI	Angiotensin receptor-neprilysin inhibitor
AUC	Area under the curve
CI	Confidence interval
EF	Left ventricular ejection fraction
EF_A	Left ventricular ejection fraction at admission
GDMT	Guideline-directed medical therapy
HFpEF	Heart failure with preserved ejection fraction
HFrEF	Heart failure with reduced ejection fraction
LVEF	Left ventricular ejection fraction
MFH	Metabolic, Functional, and Hemodynamic (staging score)
NF-κB	Nuclear factor kappa-light-chain-enhancer of activated B cells
NT-proBNP	N-terminal pro-B-type natriuretic peptide
NYHA	New York Heart Association (functional classification)
OAT	Organic anion transporter
OR	Odds ratio
ROC	Receiver operating characteristic
ROS	Reactive oxygen species
SE	Standard error
SGLT2	Sodium-glucose cotransporter-2
UA	Uric acid
ULT	Urate-lowering therapy
VIF	Variance inflation factor
XO	Xanthine oxidase
XOR	Xanthine oxidoreductase

## References

[1] de la Espriella R., Núñez-Marín G., Codina P., Núñez J., Bayés-Genís A. (2023). Biomarkers to Improve Decision-Making in Acute Heart Failure. Card. Fail. Rev..

[2] Kumrić M., Borovac J.A., Kurir T.T., Božić J. (2021). Clinical Implications of Uric Acid in Heart Failure: A Comprehensive Review. Life.

[3] Buzas R., Ivan V.-S., Gheorghe-Fronea O.-F., Morgovan A.F., Ardelean M., Albulescu N., Dorobantu M., Lighezan D.F. (2021). Arterial Hypertension and Serum Uric Acid in Elderly-SEPHAR III Study. Arq. Bras. Cardiol..

[4] Zheng Y., Chen Z., Yang J., Zheng J., Shui X., Yan Y., Huang S., Liang Z., Lei W., He Y. (2024). The Role of Hyperuricemia in Cardiac Diseases: Evidence, Controversies, and Therapeutic Strategies. Biomolecules.

[5] Buzas R., Rogobete A.F., Popovici S.E., Mateescu T., Hoinoiu T., Sorop V.B., Bratu T., Ticlea M., Popoiu C.M., Sandesc D. (2018). Nuclear Transcription Factor Kappa B (NF-κB) and Molecular Damage Mechanisms in Acute Cardiovascular Diseases. A Review. J. Cardiovasc. Emergencies.

[6] Mackenzie I.S., Hawkey C.J., Ford I., Greenlaw N., Pigazzani F., Rogers A., Struthers A.D., Begg A.G., Wei L., Avery A.J. (2022). Allopurinol versus Usual Care in UK Patients with Ischaemic Heart Disease (ALL-HEART): A Multicentre, Prospective, Randomised, Open-Label, Blinded-Endpoint Trial. Lancet.

[7] Ambrosio G., Leiro M.G.C., Lund L.H., Coiro S., Cardona A., Filippatos G., Ferrari R., Piepoli M.F., Coats A.J.S., Anker S.D. (2021). Serum Uric Acid and Outcomes in Patients with Chronic Heart Failure through the Whole Spectrum of Ejection Fraction Phenotypes: Analysis of the ESC-EORP Heart Failure Long-Term (HF LT) Registry. Eur. J. Intern. Med..

[8] Givertz M.M., Anstrom K.J., Redfield M.M., Deswal A., Haddad H., Butler J., Tang W.H.W., Dunlap M.E., LeWinter M.M., Mann D.L. (2015). Effects of Xanthine Oxidase Inhibition in Hyperuricemic Heart Failure Patients: The Xanthine Oxidase Inhibition for Hyperuricemic Heart Failure Patients (EXACT-HF) Study. Circulation.

[9] Ndrepepa G. (2018). Uric Acid and Cardiovascular Disease. Clin. Chim. Acta.

[10] Ezzat M.A.W., Boghdady A.M., Ibrahim K.F.A., Dahab L.H.A. (2019). Correlation between Serum Uric Acid Level and Left Ventricular Ejection Fraction in Patients with Congestive Heart Failure. World J. Cardiovasc. Dis..

[11] Borghi C., Rosei E.A., Bardin T., Dawson J., Dominiczak A., Kielstein J.T., Manolis A.J., Perez-Ruiz F., Mancia G. (2015). Serum Uric Acid and the Risk of Cardiovascular and Renal Disease. J. Hypertens..

[12] Saini V., Singh V.B., Srivastava M., Kumhar M., Bharatiya S. (2023). Uric Acid: New Prognostic Marker in Line for Heart Failure: A Hospital Based Cross Sectional Study. Int. J. Pharm. Clin. Res..

[13] Okazaki H., Shirakabe A., Kobayashi N., Hata N., Shinada T., Matsushita M., Yamamoto Y., Shibuya J., Shiomura R., Nishigoori S. (2016). The Prognostic Impact of Uric Acid in Patients with Severely Decompensated Acute Heart Failure. J. Cardiol..

[14] Huang G., Qin J., Deng X., Luo G., Yu D., Zhang M., Zhou S., Wang L. (2019). Prognostic Value of Serum Uric Acid in Patients with Acute Heart Failure. Medicine.

[15] Kaufman M., Guglin M. (2013). Uric Acid in Heart Failure: A Biomarker or Therapeutic Target?. Heart Fail. Rev..

[16] Shimizu T., Yoshihisa A., Kanno Y., Takiguchi M., Sato A., Miura S., Nakamura Y., Takeishi Y. (2015). Relationship of Hyperuricemia with Mortality in Heart Failure Patients with Preserved Ejection Fraction. Am. J. Physiol.-Heart Circ. Physiol..

[17] Li L., Chang Y., Li F., Yin Y. (2024). Relationship between Serum Uric Acid Levels and Uric Acid Lowering Therapy with the Prognosis of Patients with Heart Failure with Preserved Ejection Fraction: A Meta-Analysis. Front. Cardiovasc. Med..

[18] Virdis A., Masi S., Casiglia E., Tikhonoff V., Cicero A.F.G., Ungar A., Rivasi G., Salvetti M., Barbagallo C.M., Bombelli M. (2020). Identification of the Uric Acid Thresholds Predicting an Increased Total and Cardiovascular Mortality Over 20 Years. Hypertension.

[19] Kuwabara M., Niwa K., Hisatome I., Nakagawa T., Roncal-Jimenez C.A., Andres-Hernando A., Bjornstad P., Jensen T., Sato Y., Milagres T. (2017). Asymptomatic Hyperuricemia Without Comorbidities Predicts Cardiometabolic Diseases. Hypertension.

[20] Călburean P.-A., Lupu S., Huțanu A., Oprica M., Opriș D.R., Stan A., Scurtu A.-C., Aniței D., Harpa M., Brînzaniuc K. (2024). Natriuretic Peptides and Soluble ST2 Improves Echocardiographic Diagnosis of Elevated Left Ventricular Filling Pressures. Sci. Rep..

[21] Thanassoulis G., Brophy J.M., Richard H., Pilote L. (2010). Gout, Allopurinol Use, and Heart Failure Outcomes. Arch. Intern. Med..

[22] Ibănescu R., Mîțu D.-A., Goje I.-D., Goje G.-I., Lighezan D. (2025). History of Heart Failure Definition. Card. Fail. Rev..

[23] Noman A., Ang D.S.C., Ogston S., Lang C.C., Struthers A.D. (2010). Effect of High-Dose Allopurinol on Exercise in Patients with Chronic Stable Angina: A Randomised, Placebo Controlled Crossover Trial. Lancet.

[24] Kanbay M., Jensen T., Solak Y., Le M., Roncal-Jimenez C., Rivard C., Lanaspa M.A., Nakagawa T., Johnson R.J. (2016). Uric Acid in Metabolic Syndrome: From an Innocent Bystander to a Central Player. Eur. J. Intern. Med..

[25] Cicero A.F.G., Cosentino E.R., Kuwabara M., Degli Esposti D., Borghi C. (2019). Effects of Allopurinol and Febuxostat on Cardiovascular Mortality in Elderly Heart Failure Patients. Intern. Emerg. Med..

[26] Niculescu R., Stoian A., Arbănași E.M., Russu E., Babă D.-F., Manea A., Stoian M., Gliga F.I., Cocuz I.G., Sabău A.H. (2025). The Dual Role of Perivascular Adipose Tissue in Vascular Homeostasis and Atherogenesis: From Physiology to Pathological Implications. Int. J. Mol. Sci..

[27] Trimarchi G., Pizzino F., Paradossi U. (2025). Inflammation in Chronic Heart Failure: An Unsolved Puzzle. Int. J. Cardiol..

[28] Hare J.M., Mangal B., Brown J., Fisher C., Freudenberger R., Colucci W.S., Mann D.L., Liu P., Givertz M.M., Schwarz R.P. (2008). Impact of Oxypurinol in Patients with Symptomatic Heart Failure. J. Am. Coll. Cardiol..

[29] Lyu D., Zhuang R., Li J., Wu Y., Di Y., Song M., Ma L., Li J., Zhang Y. (2025). Association of Hyperuricemia with Coronary Heart Disease and Other Cardiovascular Outcomes: A Systematic Review and Dose-Response Meta-Analysis. PLoS ONE.

[30] Doehner W., Anker S.D., Butler J., Zannad F., Filippatos G., Ferreira J.P., Salsali A., Kaempfer C., Brueckmann M., Pocock S.J. (2022). Uric Acid and Sodium-Glucose Cotransporter-2 Inhibition with Empagliflozin in Heart Failure with Reduced Ejection Fraction: The EMPEROR-Reduced Trial. Eur. Heart J..

[31] Packer M. (2020). Uric Acid Is a Biomarker of Oxidative Stress in the Failing Heart: Lessons Learned from Trials with Allopurinol and SGLT2 Inhibitors. J. Card. Fail..

[32] Doehner W., Anker S.D., Butler J., Zannad F., Filippatos G., Coats A.J.S., Ferreira J.P., Henrichmoeller I., Brueckmann M., Schueler E. (2024). Uric Acid and SGLT2 Inhibition with Empagliflozin in Heart Failure with Preserved Ejection Fraction: The EMPEROR-Preserved Trial. JACC Heart Fail..

[33] Min K.H., Go A.S., Lee K., Parikh R.V., Horiuchi K.M., Ambrosy A.P., Tan T.C., Srikanth K., Hamilton S.A., Svetlichnaya J. (2025). Guideline-Directed Medical Therapy and Outcomes Among Patients with Heart Failure with Improved Ejection Fraction. J. Am. Coll. Cardiol..

[34] McDowell K., Welsh P., Docherty K.F., Morrow D.A., Jhund P.S., de Boer R.A., O’Meara E., Inzucchi S.E., Køber L., Kosiborod M.N. (2022). Dapagliflozin Reduces Uric Acid Concentration, an Independent Predictor of Adverse Outcomes in DAPA-HF. Eur. J. Heart Fail..

[35] Mogensen U.M., Køber L., Jhund P.S., Desai A.S., Senni M., Kristensen S.L., Dukát A., Chen C.-H., Ramires F., Lefkowitz M.P. (2018). Sacubitril/Valsartan Reduces Serum Uric Acid Concentration, an Independent Predictor of Adverse Outcomes in PARADIGM-HF. Eur. J. Heart Fail..

[36] Selvaraj S., Claggett B.L., Pfeffer M.A., Desai A.S., Mc Causland F.R., McGrath M.M., Anand I.S., van Veldhuisen D.J., Kober L., Janssens S. (2020). Serum Uric Acid, Influence of Sacubitril-Valsartan, and Cardiovascular Outcomes in Heart Failure with Preserved Ejection Fraction: PARAGON-HF. Eur. J. Heart Fail..

[37] Baba D.-F., Suciu H., Avram C., Harpa M.M., Stoian M., Moldovan D.-A., Huma L., Rusu G., Pal T., Danilesco A. (2024). The Impact of Heart Failure Chronic Treatment Prior to Cardiac Transplantation on Early Outcomes. Medicina.

[38] Urbanowicz T., Tomaszewska M., Olasińska-Wiśniewska A., Sikora J., Straburzyńska-Migaj E., Piecek J., Białasik-Misiorny M., Krasińska-Płachta A., Tykarski A., Jemielity M. (2024). Serum Uric Acid as an Indicator of Right Ventricular Dysfunction in LVAD Patients: A Preliminary Study. Biomedicines.

